# Impact of deliberate practice on evidence-based medicine attitudes and behaviours of health care professionals

**DOI:** 10.1007/s40037-020-00634-9

**Published:** 2020-11-26

**Authors:** Eelco Draaisma, Lauren A. Maggio, Jolita Bekhof, A. Debbie C. Jaarsma, Paul L. P. Brand

**Affiliations:** 1 Lifelong Learning, Education & Assessment Research Network (LEARN), University Medical Centre Groningen, Groningen, The Netherlands; 2 Department of Medicine, Uniformed Services University of the Health Sciences, Bethesda, USA; 3 Women and Children’s Centre, Isala Hospital, Zwolle, The Netherlands; 4 Isala Academy, Department of Medical Education and Faculty Development, Isala Hospital, Zwolle, The Netherlands

**Keywords:** Evidence-based medicine, Postgraduate medical education, Deliberate practice, Four-component instructional design model

## Abstract

**Introduction:**

Although evidence-based medicine (EBM) teaching activities may improve short-term EBM knowledge and skills, they have little long-term impact on learners’ EBM attitudes and behaviour. This study examined the effects of learning EBM through stand-alone workshops or various forms of deliberate EBM practice.

**Methods:**

We assessed EBM attitudes and behaviour with the evidence based practice inventory questionnaire, in paediatric health care professionals who had only participated in a stand-alone EBM workshop (controls), participants with a completed PhD in clinical research (PhDs), those who had completed part of their paediatric residency at a department (Isala Hospital) which systematically implemented EBM in its clinical and teaching activities (former Isala residents), and a reference group of paediatric professionals currently employed at Isala’s paediatric department (current Isala participants).

**Results:**

Compared to controls (*n* = 16), current Isala participants (*n* = 13) reported more positive EBM attitudes (*p* < 0.01), gave more priority to using EBM in decision making (*p* = 0.001) and reported more EBM behaviour (*p* = 0.007). PhDs (*n* = 20) gave more priority to using EBM in medical decision making (*p* < 0.001) and reported more EBM behaviour than controls (*p* = 0.016).

**Discussion:**

Health care professionals exposed to deliberate practice of EBM, either in the daily routines of their department or by completing a PhD in clinical research, view EBM as more useful and are more likely to use it in decision making than their peers who only followed a standard EBM workshop. These findings support the use of deliberate practice as the basis for postgraduate EBM educational activities.

## Introduction

Evidence-based medicine (EBM) is regarded as one of the most prominent milestones in modern medicine [1–4]. It has made well-recognised contributions to high-quality, individualised, cost-conscious care for patients [2, 3, 5, 6]. Worldwide, EBM principles are used in the development and application of clinical practice guidelines, which help clinicians make optimal decisions for their patients’ health issues [1].

The process of EBM comprises a series of steps: formulating a structured clinical question based on a patient scenario, systematically searching the literature for evidence, appraising the validity and the applicability of the retrieved evidence, integrating it with the physician’s clinical expertise and the patient’s values and preferences, and evaluating the process [7–9]. Most efforts of the EBM movement have been aimed at standardising and studying the first three steps of the EBM process [1, 3]. The application of these EBM steps requires sufficient knowledge about and skills in framing a clinical question, searching and appraising relevant evidence, and applying the retrieved results to the patient’s scenario [9].

EBM training programs, targeted at improving this set of knowledge and skills, are ubiquitous at both undergraduate and postgraduate levels of medical education [10, 11]. Despite this almost universal exposure of medical students and residents to EBM teaching, most doctors do not explicitly use EBM in medical decision making [3, 12, 13]. Apparently, current approaches to teaching EBM have failed to change the behaviour of its learners in clinical practice, the third level of Kirkpatrick’s four-level model of evaluation of training programmes [14]. When asked why they refrain from using EBM in their clinical decision making, physicians report a lack of EBM knowledge and skills [15, 16]. Systematic reviews of EBM teaching interventions show only small beneficial effects on EBM knowledge and skills [10, 17–19]. These observations indicate that the failure of current EBM teaching programmes to change learners’ behaviour lies at the second level of Kirkpatrick’s model, i.e. the learners’ attitudes, knowledge and skills [14].

Many physicians report a negative attitude towards EBM [3, 5, 20]. Although they agree that rigorous scientific evidence should form the basis of medical care, they struggle with how to apply this evidence in daily clinical practice [1, 4, 5]. Because of the emphasis in the EBM literature on the technical skills of searching and appraising evidence, clinicians tend to view EBM as an academic exercise, leaving limited room for integrating their clinical expertise and the patient’s values and preferences [5, 7, 20]. This suggests that EBM education should not only target learners’ EBM knowledge and skills, but also physician’s attitudes to EBM, the application of newly acquired EBM knowledge and skills in daily practice, and the effects this may have on patient outcomes.

Three issues need to be considered when thinking about designing more effective EBM education. First, it should be realised that EBM skills are complex, comprising interacting elements that are also influenced by contextual factors [10]. Ericsson’s model of acquiring competence in complex skills advocates the learning of such skills through deliberate practice, a process of systematic repeated exposure to the tasks of performing the complex skills, scaffolded by repeated, structured and constructive feedback [21]. In EBM education, this could be approached in various ways, for example by long-term, ongoing regular practice in EBM tasks with feedback from supervisors proficient in applying EBM in their daily work, or by performing clinical research projects under expert supervision over a number of years, such as in a clinical PhD research programme. Second, it has been observed that the efficacy of EBM education increases when the teaching is integrated into clinical practice, as opposed to classroom-based teaching [10, 22]. Finally, positive EBM role models in clinical supervisors are particularly strong facilitators for learners’ integration of EBM in clinical practice [20].

Members of this author team recently described how EBM was implemented into a busy paediatric department in a Dutch general teaching hospital (Isala Hospital), based on the principles of deliberate practice, integration in clinical practice, and a team-based approach [23]. This EBM programme comprised EBM training for the entire team of paediatricians and nurse practitioners (NP) in the unit, structurally embedding EBM activities in the department’s weekly routine, including all paediatricians, residents and NP in these activities, development of over 100 local evidence-based clinical practice guidelines, and continuous evaluation of the impact of these activities on patient care. The project reduced practice variation between paediatricians and was associated with a more cost-conscious approach to diagnosis by reducing the use of unnecessary diagnostics. In addition, the increasingly open discussion within the team about uncertainties and limitations of evidence-based guidelines changed the department’s culture from one of expertise (in which medical specialists were expected to have the knowledge and the answers) to a culture of growth and learning (in which all department members are encouraged to acknowledge uncertainty and ask questions) [23].

The primary aim of the present study was to explore to what extent exposure to deliberate EBM practice, either in a clinical environment set up as an EBM practice [23] or in a clinical PhD research programme, affects the learners’ EBM attitudes and behaviour. We hypothesised that health care professionals exposed to deliberate EBM practice would have a more positive attitude towards EBM and were more likely to show EBM behaviours than health care professionals who had only followed a single EBM workshop in their medical education career. The secondary aim was to explore whether the effects of exposure to deliberate EBM practice would persist after cessation of this exposure.

## Methods

### Design

This was a cross-sectional study of purposefully collected data on EBM attitudes and behaviours between paediatric health care professionals exposed to various forms of EBM education. We wanted to compare the effects of deliberate EBM practice (at Isala’s paediatric department, as previously reported [23], or by having completed a clinical PhD research programme) to exposure to a stand-alone EBM workshop, which is the current practice of EBM teaching in most Dutch postgraduate medical education (PGME) programmes including paediatrics. We limited data collection to paediatric health care professionals and to the Dutch northeast educational region, to avoid bias by differences between health care or PGME programme settings.

### Participants

#### Reference group

We considered the permanent staff members of Isala’s paediatric department the reference group of ongoing deliberate EBM practice, because they have participated actively in multiple weekly EBM activities including critically appraised topics, local guideline discussions, and journal clubs, since the inception of the department’s evidence based practice programme in 2005, or since the start of their work at Isala (for details, see [23]). At the time of data collection (January–March 2018), this *reference group *population comprised 16 paediatricians and 6 nurse practitioners (NPs).

#### Residents and recently qualified paediatricians

This study was conducted in the Netherlands’ northeast educational region, where paediatric residents receive their clinical workplace PGME at the University Medical Centre Groningen (UMCG, 36–45 months) and at one of six affiliated general teaching hospitals (15–24 months). All paediatric residents in this region attend a 2-day, classroom-based EBM course once during residency. In the UMCG and five of the six general hospitals, other EBM activities during paediatric residency are limited to incidental journal clubs and critically appraised topic (CAT) sessions, with limited participation from supervising faculty. At Isala Hospital, by contrast, paediatric residents are exposed to deliberate EBM practice with active participation in multiple weekly EBM activities (as outlined above, and in detail in [23]) for the duration of their general hospital rotation.

All paediatric residents and paediatricians who had completed the general hospital part of their PGME training in one of the six general teaching hospitals in the Dutch northeast educational region between 2006 (the year after the EBM programme at Isala’s paediatric department had started) and 2017 were considered eligible for the study and were approached to participate. We considered *former Isala residents *as participants with limited exposure to deliberate EBM practice and the other residents as *control participants,* whose only exposure to EBM teaching was a stand-alone 2‑day workshop.

#### Participants with a completed clinical PhD thesis

Competition for paediatric residency positions in the Netherlands is fierce, with a rejection rate of 90–95% of all junior doctors applying for the programme. Because research experience, particularly in a field relevant to paediatrics, increases the likelihood of acquiring a paediatric residency post, many junior doctors desiring to be paediatricians pursue a PhD research programme after graduation as doctors. Dutch clinical PhD research programmes typically take 4 years to complete. During a PhD programme, candidates follow widespread training in research methodology, clinical epidemiology, literature search and appraisal, and considering the implications of their research findings in clinical practice. We considered participants with a completed PhD thesis on clinical research as participants with 4‑year exposure to deliberate EBM practice (*PhDs*).

### Instruments and procedures

Between August 2017 and March 2018, all eligible participants as outlined above were asked to participate in the study. We used the evidence-based practice inventory (EBPi) to assess participants’ EBM attitudes and behaviours. This validated self-report questionnaire includes 26 items in five dimensions. Each item contains a statement, the agreement to which participants score on a six-point Likert scale. Higher scores indicate more positive EBM attitudes or more self-reported EBM behaviour [24], with minimally important differences between subjects ranging from 1.0 to 1.5 points for the different subscales. For the purpose of this study, participants’ scores on the dimensions ‘Attitude’ (8 items, minimum score 8, maximum score 48, assessing learners’ attitude towards EBM, and their perception of its value in increasing quality of care), ‘Decision making’ (3 items, score range 3–18, assessing priority given to using EBM principles in medical decision making), and ‘Intention and behaviour’ (4 items, score range 4–24, assessing EBM behaviour in daily practice) were analysed to represent participants’ EBM attitudes and behaviour. We also collected demographic and other background information by questionnaire.

Questionnaires were administered to eligible residents on paper during a (non-EBM related) regional teaching session. Other eligible subjects (i.e. residents who did not attend the teaching session and recently qualified paediatricians) were invited by e‑mail to complete the questionnaires digitally. The reference group of Isala paediatricians and NPs were administered the questionnaires on paper, along with the request to complete and return them within one month.

Participation was voluntary, and all participants provided written informed consent. After collection, the data were anonymised for the purpose of analysis. The study was approved by Isala’s medical ethical review committee (file no. 2018-47).

### Statistical analysis

Data were analysed using SPSS (IBM SPSS Statistics version 23) and GraphPad Prism 7, using standard parametric analysis (one-way analysis of variance [ANOVA]) after testing for normal distribution. A significance level (α) of 0.05 was used for all analyses.

## Results

Of the 62 eligible subjects, 50 (81%) participated: 14 former Isala residents (28%), 20 PhDs (40%), and 16 controls (32%). Of the 22 subjects in the eligible current Isala group, 13 (59%) participated (11 paediatricians and 2 NPs). Three subjects from the current Isala group had completed a clinical research PhD, a number considered too small to analyse as a separate subgroup.

Characteristics of study participants are presented in Tab. 1, along with their EBPi scores. There were no significant differences in EBPi scores between participants who completed the questionnaire on paper or digitally.

**Table 1:** Demographics and EBPi scores of study groups

Group	*n*	Age in years, mean (SD)	Female gender (%)	EBPi score ‘attitude’, mean (SD)	EBPi score ‘decision making’, mean (SD)	EBPi score ‘intention and behaviour’, mean (SD)
Controls	16	31.6 (1.8)	13 (72)	34.74 (4.87)	10.47 (2.00)	13.35 (3.39)
Former Isala	14	32.2 (3.5)	13 (93)	35.71 (4.32)	11.29 (1.54)	14.29 (2.87)
PhDs	20	35.5 (3.3)	13 (65)	36.95 (3.66)	13.00 (1.69)	16.15 (3.75)
Current Isala (reference group)	13	49.7 (7.9)	6 (46)	39.42 (6.29)	13.25 (1.86)	16.92 (3.40)

### EBM attitude, decision making and behaviour

**Fig. 1:**
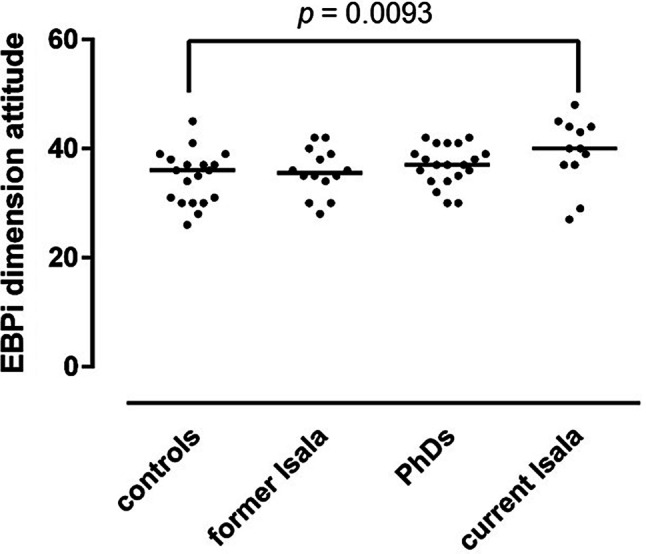
EBPi ‘attitude’ dimension scores, reflecting participants’ attitude towards EBM, in study groups exposed to standalone EBM workshop only (controls), 15–24 months of exposure to deliberate EBM practice during residency training (former Isala), 4‑year intensive and active exposure to deliberate EBM practice in clinical PhD research programme (PhDs) and the reference group of ongoing long-term daily exposure to deliberate EBM practice (current Isala). The *p* value reflects a post-hoc between-group comparison (one-way ANOVA)

**Fig. 2:**
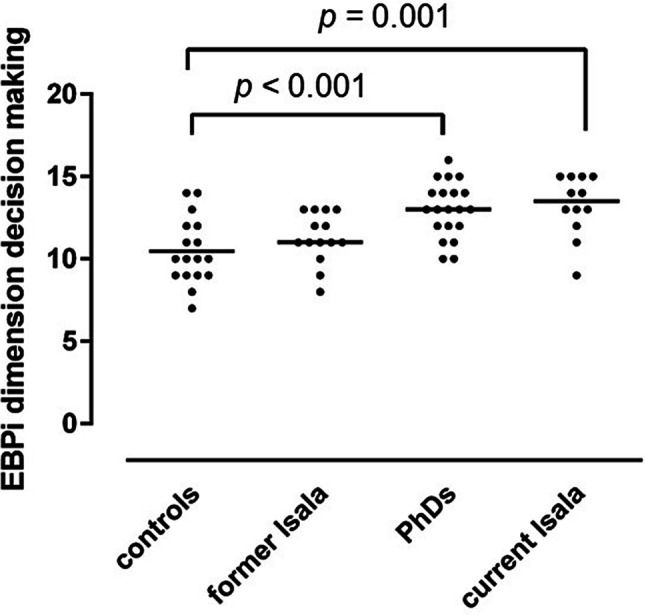
EBPi ‘decision making’ dimension scores, reflecting the priority participants give to using EBM in their clinical decision making, in study groups exposed to standalone EBM workshop only (controls), 15–24 months of exposure to deliberate EBM practice during residency training (former Isala), 4‑year intensive and active exposure to deliberate EBM practice in clinical PhD research programme (PhDs) and the reference group of ongoing long-term daily exposure to deliberate EBM practice (current Isala). The *p* values reflect post-hoc between-group comparisons (one-way ANOVA)

**Fig. 3:**
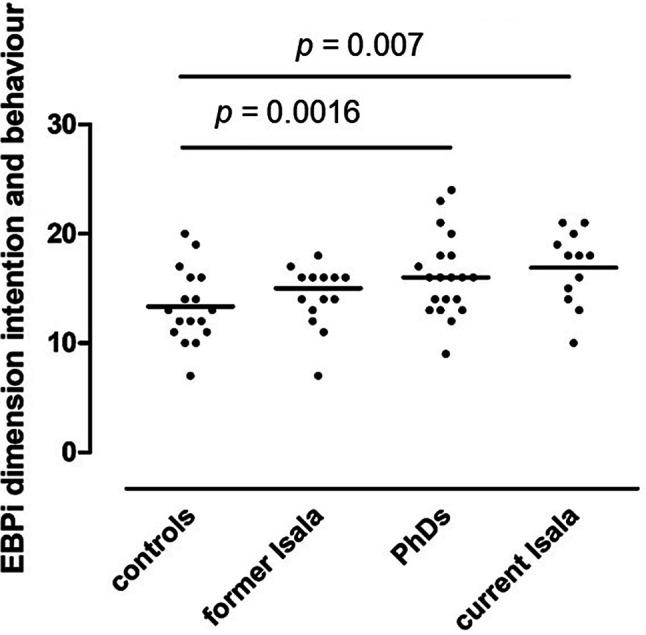
EBPi ‘intention and behaviour’ dimension scores, reflecting the participants’ self-reported EBM behaviour in daily practice, in study groups exposed to standalone EBM workshop only (controls), 15–24 months of exposure to deliberate EBM practice during residency training (former Isala), 4‑year intensive and active exposure to deliberate EBM practice in clinical PhD research programme (PhDs) and the reference group of ongoing long-term daily exposure to deliberate EBM practice (current Isala). The *p* value reflects a post-hoc between-group comparison (one-way ANOVA)

Mean scores, with standard deviations, on the EBPi dimensions ‘attitude’, ‘decision making’ and ‘intention and behaviour’ are presented in Tab. 1 and in Figs. 1, 2 and 3. Compared to controls, the reference group of current Isala participants reported a significantly more positive attitude towards EBM (*p* = 0.0093) (Fig. 1). Both PhDs and current Isala participants reported significantly higher priority given to using EBM principles in medical decision making (*p* ≤ 0.001) (Fig. 2) and reported using significantly more EBM behaviour in daily practice (*p* < 0.02) (Fig. 3).

## Discussion

The results of this study show that, compared to a standard stand-alone 2‑day EBM workshop, exposure to deliberate EBM practice is associated with participants giving significantly more priority to using EBM principles in medical decision making, and showing more EBM behaviour in daily practice. The reference group of paediatricians and NPs with ongoing deliberate EBM practice including active involvement in numerous weekly EBM tasks and activities also showed a more positive attitude towards EBM than the control group of paediatric residents and recently qualified paediatricians who had followed only the stand-alone EBM workshop. These findings suggest that health care professionals exposed to deliberate practice of EBM, either in the daily routines of their PGME training department or by completing a PhD in clinical research, view EBM as more useful and are more likely to use it in decision making than their peers not exposed to deliberate practice of EBM.

Previous studies showed that a stand-alone EBM training can improve short-term knowledge and skills, but little is known about its impact on clinicians’ attitudes and behaviours [12, 19]. According to Ericsson’s theory of deliberate practice, competence in and application of complex skills can be increased by spending more time on practice, accompanied by immediate feedback [21]. Support for this theory has been found in relation to surgical [25] and communication skills [26–28], which improve with repeated practice followed by structured and constructive feedback. We considered that this theory would also apply to the complex skills involved in the application of EBM, and might help in understanding why stand-alone EBM education has limited effects on learners’ application of this knowledge and these skills in clinical practice [3, 12, 13]. Although the effects were small, they exceeded the minimally important differences of 1.0–1.5 points for the different EBPi subscales [24, 29]. Our results align with deliberate practice theory whereby repeated and ongoing exposure to EBM tasks and activities may contribute to developing more positive attitudes towards and greater use of EBM in practice.

Although the theory of deliberate practice appears useful in understanding the learning of complex skills in medical education, including the skills involved in the application of EBM, the elaboration of this theory into concrete EBM teaching activities in PGME supporting the uptake and use of EBM by residents remains to be further established. Educational scientists have argued that the teaching of complex cognitive tasks requires instructional approaches like the Four Component Instructional Design (4C/ID) model [30], and adoption of this model has been proposed as a useful guide for designing EBM teaching activities [22]. According to this model, learning tasks are presented both as whole-task and part-task activities. Whole-task approaches introduce learners to the combined steps of an activity upfront and ask them to practice all of the requisite skills as a cohesive activity, with graded levels of task complexity and support depending on the learner’s performance. These are supplemented with cognitively less challenging part-task activities, which help novice learners to build confidence and competence towards completing the whole complex task [22]. The design and activities of the Isala EBM implementation programme reflect components of the 4C/ID model. By integrating EBM activities into the department’s weekly routine of meetings and teaching sessions, paediatricians, NPs and residents have the opportunity to practice both whole- (e.g., CAT) and part-task activities (e.g., journal club), supported by information on the department’s intranet [23]. With supervising faculty not only attending, but also preparing and facilitating these meetings, and participating in performing the complex tasks, they continue to practice these complex skills themselves, and serve as a role model supporting the residents in their EBM learning. This team-based role modelling has been recognised as one of the key success factors in the implementation of EBM in teaching departments [20], and has been associated with improved patient outcomes as compared to departments without an integrated and structured EBM programme [5].

The feedback and mentoring from supervising staff on the EBM activities is partly dependent on the individual staff members’ EBM expertise and attitude, which varies considerably, given the wide scatter of EBPi scores (Figs. 1, 2 and 3). Further work is needed to explore the impact of consistent EBM role modelling and feedback on EBM activities from supervising staff on the development of residents’ EBM attitudes and behaviours in PGME.

There were no significant differences in EBPi scores between the former Isala residents who had been exposed to deliberate EBM practice for 15–24 months during their paediatric PGME training and their peers who had only followed a stand-alone 2‑day EBM workshop (Tab. 1; Figs. 1, 2 and 3). The benefits of involvement in deliberate practice of EBM therefore seem to decline after active engagement in the programme is discontinued. Such deterioration of learning effects without ongoing practice has been observed in other complex medical skills, such as airway management and surgical skills [31, 32]. Conversely, the longer, more intense, and full-time exposure to deliberate practice during a 4-year clinical PhD research programme was associated with more persistent priority given to EBM in clinical decision making and applying more EBM behaviour in practice than only following a stand-alone EBM workshop (Figs. 2 and 3). This suggests that exposure to deliberate EBM practice requires a certain threshold of time and intensity to achieve long-term effects on EBM attitudes and behaviour. In addition, maintaining a positive attitude towards EBM appears to be dependent on ongoing exposure to deliberate EBM practice (Fig. 1).

This study presents the first evaluation of the role of deliberate practice in the development of health care professionals’ EBM attitudes and behaviour during PGME and subsequent independent practice, using a validated questionnaire. Several limitations of the study need to be considered. First, the study groups differed in more characteristics than only exposure to deliberate EBM practice. Specifically, whilst the other groups consisted of paediatric residents and recently qualified paediatricians, the reference group of current Isala workers consisted largely of paediatricians with considerably greater clinical experience, as illustrated by the age difference between the groups (Tab. 1). Furthermore, the observational, cross-sectional design leaves room for other, unknown differences between groups which may have affected outcomes. We believe it is unlikely, however, that the difference in age and experience between the reference group of current Isala workers and the other groups is responsible for our main study findings, as previous studies have consistently shown that even highly experienced clinicians show very little application of EBM skills and techniques in their daily clinical practice [12, 13, 16, 33]. In addition, the higher EBM priority and self-reported EBM behaviour in the other deliberate practice group of PhDs also suggest that deliberate EBM practice is a more likely explanation of the differences that we observed between the study groups than age or clinical experience. Another limitation was the small number of eligible participants, limiting the study’s power to detect potentially relevant differences between study groups. Because the EBPi relies on self-report, no inferences can be made about participants’ displayed EBM behaviour in practice.

Stand-alone, classroom-based courses and workshops have been the mainstay of postgraduate EBM education for decades [10, 34]. This strategy has been largely ineffective in improving clinicians’ EBM attitudes and behaviours [12, 20]. The results of this study suggest that education based on the theory of deliberate practice offers an alternative which might achieve the latter, as it has in surgical and communication skills [25–28]. Full application of the 4C/ID model may help to increase the educational impact of deliberate EBM practice training in PGME. Future studies should evaluate the effects of such training, among larger samples and applying prospective methodology.
